# Catatonia Presentation as a Rare Alcohol Withdrawal Symptom in a Patient With No Past Psychiatry History: A Case Report

**DOI:** 10.7759/cureus.13722

**Published:** 2021-03-05

**Authors:** Stanley Nkemjika, Olaniyi Olayinka, Gulshan Begum, Tolu Olupona, Ayodeji Jolayemi

**Affiliations:** 1 Public Health/Epidemiology, Georgia State University, Atlanta, USA; 2 Psychiatry and Behavioral Sciences, Interfaith Medical Center, Brooklyn, USA

**Keywords:** catatonia, alcohol withdrawal syndrome, psychosis

## Abstract

A complicated alcohol withdrawal syndrome (AWS) includes epileptic seizures and/or delirium tremens (DT). However, there is still a dearth of literature for catatonia as a consequence of AWS especially in terms of clinical reports. Secondly, the few noted reported cases in the literature were mainly of non-American populations. Hence, we present the case of a middle-aged woman with no past psychiatric history admitted for psychosis and altered sensorium with delayed catatonic features in the context of a history of alcohol use disorder. Ms. M., a 44-year-old African American female with no past psychiatric history but a past medical history of gastric bypass surgery, presented to the psychiatric emergency department via emergency medical service due to roaming the street because of acute onset of altered mental status and psychotic features. She had a Clinical Institute Withdrawal Assessment of Alcohol Scale (CIWA) score of 33 following last alcohol use a few hours prior to presentation. While on the inpatient unit, the patient had an isolated episode of catatonic stupor despite being administered lorazepam 2mg every four hours as needed. Supportive medical staff should also be aware of catatonia as a rare manifestation of alcohol withdrawal. A persistent, thorough medical workup and evidence-based "investigative" history gathering can help elucidate the source of the presenting symptom in this patient population.

## Introduction

Catatonia is a complex neuropsychiatric syndrome that has been described in medical literature since the sixteenth century. Life-threatening complications have been reported in literature [1]. Documented evidence suggests that the etiology of catatonia is still unclear [2] as literature depicts more occurrences in patients with previous psychiatric histories like affective disorders and psychotic disorders. Etiologically, apart from psychiatric disorders, medical causes of catatonia like neurological and metabolic diseases are common [3]. Among substances, alcohol and benzodiazepine withdrawal have been reported to be associated with catatonia. Though reported catatonia has been elicited in laboratory settings among animals, only a few such cases have been reported to be attributable to alcohol use [4]. Rather, there seems to be more available literature on catatonia symptoms from benzodiazepine withdrawals [5] than substance use-induced origin. Thus, catatonia could be implied as being more common with underlying neurologic conditions and benzodiazepine interactions. However, there is still a dearth of literature describing the relationship between alcohol use disorders and catatonia. Secondly, the few noted reported cases in the literature were mainly of populations other than North America. Hence, we present the case of a middle-aged woman with no past psychiatric history admitted for psychosis and altered sensorium with a delayed catatonic feature in the context of a history of alcohol use disorder. We propose that her alcohol use could be a factor contributing to the onset of delayed-onset catatonia.

## Case presentation

We present this case of a 44-year-old African-American female with no past psychiatric history but past medical history of gastric bypass and uterine fibroid surgery, who presented to the psychiatric emergency department via emergency medical service due to roaming the street because of acute onset of altered mental status and psychotic features. She exhibited poor reality testing, poor insight, and judgment at the time of evaluation. She was a poor historian. She reported feeling anxious and "having a panic attack" as she was afraid of being lost in the city. The patient exhibited disorganized thought processes which depicted circumstantiality and associative looseness. The patient denied auditory/visual hallucinations, thought insertion, thought broadcasting, and thought withdrawal. She denied anhedonia, feelings of hopelessness or helplessness, changes in concentration, appetite, or change in weight. She reported daily use of over-the-counter sleeping pills for one month in addition to a chronic history of "drinking and smoking marijuana" because of sleeping difficulty. Her alcohol use had lasted for more than two decades. Her baseline alcohol intake was reported at approximately 750ml of whiskey on daily basis, equivalent to 374g per day of alcohol. She reported alternating occasionally with 1.5l of red wine, equivalent to approximately 224g per day of alcohol. The patient ingested 748g of alcohol a night prior to presentation, but the alcohol use history was slightly unclear due to her presentation and her documented residential address which was 3000 miles away. The patient’s TWEAK scoring scale revealed that the patient had a score of 5 and a Clinical Institute Withdrawal Assessment of Alcohol Scale (CIWA) score of 33 which depicts problematic alcohol use. The CIWA criteria that were met include intermittent nausea with dry heaves (+4), moderate tremors with the patient's arms extended (+4), more severe sweating symptoms (+2), more severe anxiety symptoms (+5), more severe agitation symptoms (+3), severe hallucinations (+5), moderately severe hallucination (+4), mild headache (+2) and disoriented to place (+4). Her laboratory screening showed 53 IU/L aspartate aminotransferase test (AST) level (reference level: 5-34 IU/L) on presentation. She was started on risperidone 1mg PO BID for psychosis and trazodone HCL 50mg PO HS for insomnia. She was also started on folate, Vitamin B12, and lorazepam (both PRN and routine) medications.

She was subsequently admitted to the psychiatry inpatient floor for stabilization and continuing psychiatric care. Her condition had been ongoing for six months prior to presentation as she reported experiencing blackout episodes and losing her personal items on a recurrent basis. Given the patient’s psychiatric and medical history, an integrated care approach was adopted in the inpatient ward. The team considered the differential diagnosis of severe alcohol use disorder and moderate major depressive disorder. Her medication regimen was adjusted by initiating escitalopram 10mg PO daily for depressive symptoms, risperidone was tapered off while commencing aripiprazole 2mg PO daily for psychosis and augmentation of the antidepressant, hydroxyzine 25mg HS PO PRN for anxiety, lorazepam 2mg HS PO PRN for alcohol withdrawal symptoms and naltrexone 50mg PO daily for alcohol cravings. The patient was educated and advised on medication compliance. She was also educated on the benefits of individual, group, and milieu therapy for which she verbalized understanding. For the first few days, the patient’s clinical conditions improved as she was noted to be coming out of her room more often, attending to some of the group therapy sessions, and started relating with her peers. She also reported that her sleeping improved.

However, on day 3 of admission, she exhibited a transient event of catatonia which manifested as standing on the exit door of her room, not talking or responding to any stimulus. She was selectively mute and not responding to any directions. This episode resolved without any further intervention apart from the administration of IM lorazepam 2mg once. She was re-evaluated and monitored on the floor, with uncertainty whether her symptoms were as a result of alcohol withdrawal with catatonic features or an underlying neurologic condition. Hence, she was administered the Bush Francis Catatonia Rating Scale (BFRCS) which showed a screening score of 9. The severity score for the BFCRS is 22. Based on other attributable histories of presenting illnesses, which included poor self-image from recurrent gastric surgery, impulsivity (quitting her job and traveling away from her home city), and a brief psychotic episode under stress, the management team also screened the patient for borderline personality disorder; she scored 2/10 for the Maclean screening instrument for borderline personality disorder (i.e. had at least two other problems with impulsivity; made desperate efforts to avoid feeling abandoned or being abandoned). A routine computed tomography (CT) scan of the head to rule out any morbid causality returned normal.

She tolerated all her medications and began to show clinical improvement of symptoms by day 4 of her hospital stay. She began to interact more with others on the unit as she participated in supportive and group therapy sessions, attending a total of 12 group sessions during this admission. She maintained good behavioral control on the inpatient unit and had a good rapport with his peers and staff. She did not require PRN IM medication for the rest of her inpatient admission. She was motivated to continue with her mental health care as a psychiatry and rehabilitation outpatient for her alcohol use disorder as she was discharged back to the community.

## Discussion

Considering that this patient presented with no documented past psychiatric history, co-morbid obesity and past gastric bypass surgery, acute psychotic features and a CIWA score of 33 following last alcohol use of few hours prior to presentation, the effectiveness of benzodiazepines is exemplified in this case by halting catatonic episode 72 hours after presentation. Based on this clinical vignette, it is important for both emergency and inpatient services personnel to understand that catatonia is a rare clinical symptom of AWS which could manifest as a lack of interaction with the environment accompanied with specific motor symptoms. These motor symptoms vary but might include bizarre posturing and maintenance of awkward postures into which the patient is placed (waxy flexibility) [6]. Catatonic patients might also offer purposeless resistance to commands with repetitive and meaningless gestures. Based on the mix of behaviors observed in our patient, the presence of waxy flexibility, her inability to respond to any command nor any stimuli and not following appropriate eye-tracking movements is in keeping with the DSM 5 criteria for catatonia [7].

Evidence from literature has suggested that AWS-related catatonia probably represents a type of alcohol-related delirium as opposed to its own distinct disease process [8]. Hence, the difficulty with its classification and accommodation into the DSM classification and often treated as a symptomatic component of a broader alcohol-related diagnosis. The perceived support for this comes from the observation that both AWS-related catatonia and alcohol-related delirium occur three to six days from the first day of abstinence, responsive to benzodiazepines, and have no characteristic EEG readings associated or attributable to catatonia [2]. 

Catatonia is a common manifestation in patients with structural neurologic conditions, physiologic neurologic conditions (like Parkinson’s disease and epilepsy), abnormal metabolic states, psychiatric conditions (like mood disorders, psychosis, and pervasive developmental disorder) and different drug-related conditions [9]. Due to a lack of any history of organicity with her presentation, the general mental state assessment and detailed neurological examination both at the ED and inpatient unit ruled out most of the common organic conditions. However, neuroimaging requested for this case was to rule out radiologically, any index of suspicion for other organic factors that may precipitate catatonia other than AWS despite adequate medication protocol. This practice is not a routine in the management process of AWS diagnosis but was necessary considering the nature and sequence of catatonic symptom manifestation. However, the review of the neurological team following neuroimaging results yielded no organic cause of the presentation. Hence, it was vital to ensure neuroimaging which assists to rule out other likely non-psychiatric differentials that the team had in mind.

Despite the reported and little evidence-based information in the literature on both benzodiazepine and alcohol withdrawal, there is a dearth in the literature on AWS catatonic symptoms in patients globally. Based on our review of literature, there is no known reported case of AWS-related catatonia presentation in patients with no positive family history of mental disorder. Similarly, the co-morbidity in this patient is also novel and will bridge the gap in literature, especially with medical comorbidities. Additionally, there is a handful of reported studies with patients of Indian heritage [10]. Till date, there is none described among Americans across all races, ethnicity, and gender. It is worth mentioning that few reports in the literature are mainly focused on combination withdrawal of both benzodiazepines and alcohol at the same time or an imbalance in one of them during management. Similarly, considering the few case reports or original studies on catatonia, most of them are based on patients with prior history of psychiatric or family history of mental disorder. Additionally, other relationships of catatonia-related symptoms have been described in patients with a childhood history of seizure. 

Being that AWS is a medical emergency, patients with no psychiatric history presenting to the ED or being managed in the inpatient services with these symptom constellations and inadequate/inconsistent history of personal information (HPI) need to be treated with a high index of suspicion. Supportive medical staff should also be aware of catatonia as a rare manifestation of AWS and its presentation may be delayed just like our patient in this case report. A persistent, thorough medical workup and evidence-based ‘investigative’ history gathering can help elucidate the source of the presenting symptom in this patient population. In this case report, we also highlight the benefits of utilizing disease diagnostic instruments like Busch Francis Catatonia Scale to both ascertain clarity to psychiatric diagnoses of patients with no past psychiatric history and rare symptomatic presentation like catatonia [11]. This is a valuable tool to use in order to rule out other possible differential diagnoses. Hence, we highly recommend that clinical care management teams should adopt its use in clinical practice.

Notably, earlier recognition and intervention of AWS mitigates the perceived mortality rate of the condition as evidence in literature reports about 1-4% reduction. Documented interventional management is mainly pharmacology-based as aggressive initial dosing and symptom-triggered use of benzodiazepines, alongside supportive care and adequate fluid resuscitation have been beneficial. This case report serves to highlight less common and less well-studied complications of AWS which are life-threatening. Additionally, this case also illustrates the need for a high index of suspicion for patients with alcohol use disorder (AUD) in withdrawal irrespectively of benzodiazepine treatment regimen (both routine and PRN) included in the care management. The delay in the manifestation of the catatonic symptom as seen in this case report also suggests the need for continued monitoring and anticipatory alertness of health care providers in order to provide the needed intervention.

## Conclusions

We present an important finding that contributes to the body of evidence in literature in relation to AWS symptomatic presentations. The unique nature of this patient's isolated catatonic presentation, days after admission treatment, and the subsequent management protocol, especially as it pertains to the socio-demographic history of this patient with no past family history of psychiatric diagnosis is novel. Hence, health care personnel should adopt pro-active measures with regards to the emergence of catatonia within 72 hours into alcohol withdrawal as an important clinical feature of AWS, as this will ensure the timely administration of lorazepam.
